# Supplementary material for: Dynamic reflections of multidimensional health poverty in Pakistan
Source: PLoS One. 2021 Nov 5;16(11):e0258947. doi: 10.1371/journal.pone.0258947 (PMC8570466; doi:10.1371/journal.pone.0258947)
Supplement: Attachment — Submitted filename: Review report _PONE-D-21-09200.docx [file pone.0258947.s001.docx]

**Title:** Dynamic Reflections of Multidimensional Health Poverty in Pakistan

#### Manuscript ID: PONE-D-21-09200

#### Referee Recommendation: MINOR REVISIONS

I appreciate the author(s) for conducting this study on multidimensional health poverty in Pakistan via a new and novel way, especially the inter-provincial comparison. However, I have noted some issues which may render further quality to the paper, as following:

**Comments:**

1. I would suggest the author to cite most recent literature.
2. I would like to ask why equal weighting technique has been adopted instead of unequal?
3. How the cutoff decision has been made. Please mention the reference.
4. I would suggest the author to mention the hypothesis in revised manuscript.
5. Briefly define health poverty in introduction section to enhance the clarity for readers.
6. Limitations of this study should be incorporated in revised manuscript.
7. How this study is different from Iqbal & Nawaz (2017) study as it seems the replication of that study!
8. Individuals’ health poverty is directly judged by health status of individuals then why such an important indicator has not been addressed in this study?
9. Different dimensions like expenditure on food and sanitation cannot have equal importance in determining individuals’ health then why these two are equally weighted?
10. What is the justification of using Shapley approach? And not been defined theoretically.
11. It is suggested to explain dimensional decomposition.
12. Policy recommendation section needs improvement.
13. Page # 3, Line# 2: Change the word effect to affect.
