# Supplementary material for: Dynamic reflections of multidimensional health poverty in Pakistan
Source: PLoS One. 2021 Nov 5;16(11):e0258947. doi: 10.1371/journal.pone.0258947 (PMC8570466; doi:10.1371/journal.pone.0258947)
Supplement: Attachment — Submitted filename: Annotative report_ PONE-D-21-09200.docx [file pone.0258947.s002.docx]

**Annotated Report: PONE-D-21-09200**

Title of the Paper: **Dynamic Reflections of Multidimensional Health Poverty in Pakistan**

| **S. No.** | **Comments** | **Response** | **Pg#** |
| --- | --- | --- | --- |
| **Reviewer-1** | | | |
| 1 | Add the most recent reviews. | Incorporated as suggested accordingly. | 6 |
| 2 | Why equal weighting technique has been adopted instead of unequal? | In the absence of strong literary evidence the researcher is allowed to opt the equal weighting strategy and similar formation has been adopted by earlier author like Iqbal & Nawaz , (2017). | 9 |
| 3 | Define health poverty in introduction section | It has been described in different spots in literature review that’s why not included in introduction to avoid the replication. | 3 |
| 4. | Mention the hypothesis. | Hypothesis is the optional part of the research, however, the null hypothesis of the study based upon the fact that the selected set of covariates correlates does not affect the multi dimensional health poverty, whereas alternative hypothesis is otherwise. | 6 |
| 5. | How the cut-off decision has been made mention the reference. | The intuitive cut off strategy has been adopted by as proposed by  Alkire and Foster (2007), Alkire and Santos (2010) and Alkire and Housseini (2014) | 7 |
| 6. | Adopt equal weighting strategy | Equal weighting strategies has been adopted as suggested. | 9 |
| 7. | Mention limitations of the study. | The study in hand is entirely based upon HIES and PSLM data, despite the fact many other health aspects may disturb the magnitude of multidimensional health poverty. | 14 |
| 8. | How this study is different from Iqbal & Nawaz (2017) study? | Though the study in hand is based upon HIES AND PSLM data but it is extended to the recent data along with the unique set of data. | 5 |
| 9. | Individual’s health poverty is directly judged by health status of individuals why such an important indicator has not been addressed? | Because of the limited information in the existing data the household health poverty has been estimated as a representative of individual health status. | 4 |
| 10 | Different dimensions like expenditure on food and sanitation cannot have equal importance in determining individuals health then why there two are equally weighted? | These aspects has been taken as an indicator to present the nutritional deprivations of individuals. | 8 |
|  | What is the justification of using Shapley approach? And not been defined theoretically. | Justified the Shapley approach accordingly and added literary justification. | 11 |
| 11 | It is suggested to explain dimensional decomposition. | Incorporated as suggested. | 11 |
| 12 | Policy recommendation section needs improvement. | Incorporated as suggested. | 14 |
| 13 | Sequence of the study should be like this; (1) Introduction, (2) Literature, (3), Methods, (4) Results, (5) Discussion, (6) Conclusion, (7) Implications, (8) Limitations, (9) Recommendations. | Incorporated as suggested. | 1-15 |
| **Reviewer-2** | | | |
| Abstract | Revise the abstract and cover it with a theme reflecting the central idea of the study. The abstract is the face of main study. It must be high quality. Make improvement and fix typo errors. | Revised accordingly and improved the typo mistakes. | 1 |
| Introduction section | The introduction needs improvement. Discuss research gaps identified from the literature. In my opinion, points could very interesting in this study. How can social media play a leading role in educating people about this health issue? How CSR and knowledge sharing can improve the situation during the COVID-19 challenging situation? | Incorporated as suggested. | 3 |
| Methods section | Make a proper heading of the method section and explain it. I suggest adding demographic table by covering education level, age, income level and regions. See the suggested study and improve your study. See these studies to improve your work and follow them in the methods and results sections. | Improved accordingly. | 6-9 |
| Results section | See the suggested study and improve your results section. You can add graphical presentation of your findings. See these studies to improve your study. | Reviewed and incorporated the suggested studies. | 10-13 |
| Discussion section: | Make some improvement in the discussion section. It should be around one page and a half. Make it strong. See the recommended studies, and improve your sections. | Improved accordingly. | 12-13 |
| Conclusion | Make a separate heading for conclusion and do not mix it with the discussion section. Your conclusion should be based on minimum 500 words. | Corrected as per suggestions. | 13 |
| Implications | Make a separate heading for implications of your study. I suggest adding implications heading and briefly explain it in this section. | Corrected as per suggestions. | 15 |
| Limitations | Make a proper heading and discuss it adequately. | Corrected in the light of suggestions. | 14-15 |
